# Fragment-based drug discovery for disorders of the central nervous system: designing better drugs piece by piece

**DOI:** 10.3389/fchem.2024.1379518

**Published:** 2024-04-18

**Authors:** Bill W. G. L. Chan, Nicholas B. Lynch, Wendy Tran, Jack M. Joyce, G. Paul Savage, Wim Meutermans, Andrew P. Montgomery, Michael Kassiou

**Affiliations:** ^1^ School of Chemistry, The University of Sydney, Sydney, NSW, Australia; ^2^ CSIRO Manufacturing, Clayton South, VIC, Australia; ^3^ Vast Bioscience, Brisbane, QLD, Australia

**Keywords:** fragment-based drug discovery, central nervous system, Alzheimer’s disease, Parkinson’s disease, schizophrenia, Huntington’s disease, neuroinflammation, glioblastoma

## Abstract

Fragment-based drug discovery (FBDD) has emerged as a powerful strategy to confront the challenges faced by conventional drug development approaches, particularly in the context of central nervous system (CNS) disorders. FBDD involves the screening of libraries that comprise thousands of small molecular fragments, each no greater than 300 Da in size. Unlike the generally larger molecules from high-throughput screening that limit customisation, fragments offer a more strategic starting point. These fragments are inherently compact, providing a strong foundation with good binding affinity for the development of drug candidates. The minimal elaboration required to transition the hit into a drug-like molecule is not only accelerated, but also it allows for precise modifications to enhance both their activity and pharmacokinetic properties. This shift towards a fragment-centric approach has seen commercial success and holds considerable promise in the continued streamlining of the drug discovery and development process. In this review, we highlight how FBDD can be integrated into the CNS drug discovery process to enhance the exploration of a target. Furthermore, we provide recent examples where FBDD has been an integral component in CNS drug discovery programs, enabling the improvement of pharmacokinetic properties that have previously proven challenging. The FBDD optimisation process provides a systematic approach to explore this vast chemical space, facilitating the discovery and design of compounds piece by piece that are capable of modulating crucial CNS targets.

## Graphical Abstract

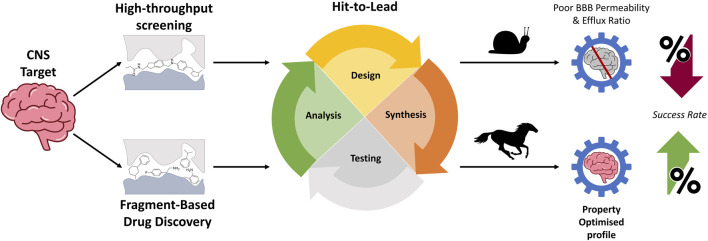

## 1 Introduction

### 1.1 CNS drug discovery

Drug discovery is an extremely expensive process, both in terms of the time and money invested. From target discovery to regulatory approval, an FDA-approved drug will take an average of 12–15 years (Brown et al., 2022) and up to US$ 3.2 billion (DiMasi et al., 2016). Whilst drug discovery is a high-risk venture with no guarantee of producing a return on any resources invested, it is still a necessary investment for improving our quality of life. Drug discovery is increasingly more important, given the ageing global population and an increasing prevalence of neurodegenerative and neuropsychiatric diseases as well as brain cancers. There are currently no cures, and the limited therapeutics available are largely ineffective; new treatments are, therefore, urgently needed. Drug developments that target disorders associated with the central nervous system (CNS) are considerably more difficult than diseases of the periphery. The CNS is protected by the blood–brain barrier (BBB), which dictates the necessary pharmacokinetic (PK) properties a drug must possess in order to enter the brain. As most CNS drug leads fail to pass through the BBB, identifying a high-quality hit with a favourable PK profile may potentially save both time and resources, making it one of the most vital steps in a drug design process. Traditional hit identification strategies have varied but ultimately see limited success in tackling issues faced in CNS drug development (Gribkoff and Kaczmarek, 2017). Therefore, exploring alternative approaches to drug development should be considered to improve the development of effective therapeutics for these diseases.

### 1.2 Challenges with conventional hit discovery and lead optimisation

Many drug discovery campaigns aim to develop orally administered drugs as they are non-invasive, convenient, and generally meet high patient acceptance (Alqahtani et al., 2021). A standard drug development program begins with the identification of “hits”. A hit molecule is a compound that has the desired activity at the target of interest; a good hit is considered one that generally obeys Lipinski’s Rule of Five (Ro5) for oral bioavailability (Hughes et al., 2011). The Ro5 advises that a molecule should have no more than five hydrogen bond donors (HBD), no more than ten hydrogen bond acceptors (HBA), a molecular weight (MW) less than 500 Daltons, and a LogP less than 5 (Lipinski et al., 2001). Although this “rule” has successfully provided guidance to many drug discovery campaigns, strictly following these could limit the design of small-molecule drugs for easily druggable targets (Hartung et al., 2023). This is emphasised by the oral drugs that had been approved between 2018 and 2022, where many of these molecules possessed high MW and number of HBA. Furthermore, the recent discovery of PROTACs also highlights that MW does not always directly correlate with oral delivery as these molecules possess MW as high as 1,000 Da and are orally bioavailable.

The most common strategy for identifying these hit molecules is through high-throughput screening (HTS), which is often used by big pharmaceutical companies for small-molecule drug development (Blay et al., 2020). A standard HTS campaign will screen a compound library (comprising hundreds of thousands of molecules) in a series of biochemical assays to identify hits (Ver Donck et al., 2020). Large libraries that contain a broad variety of structural types are necessary to explore diverse chemical space. Despite the extensive number of molecules screened, the hit rates are generally low, and the few compounds that are identified can possess suboptimal physiochemical properties. Even with a judicious selection of compound libraries that are Ro5-compliant, optimisation of the resulting hit can still be difficult. One of the major challenges when commencing a drug discovery campaign based on a hit molecule derived from conventional screening methods is that they are often complex and possess a high molecular weight (MW) (Volochnyuk et al., 2019). A standard HTS library can comprise compounds with average MWs of 400 Da. Optimising hits often involves introducing desirable functionalities which generally only adds further to the already large MW (Bienstock, 2011). Consequently, this increase in MW may compromise the oral bioavailability of the hit, as molecules larger than 400 Da typically do not undergo passive diffusion through the BBB and are also more susceptible to efflux processes (Mikitsh and Chacko, 2014; Pardridge, 2020). Furthermore, the high complexity of the initial hit poses synthetic challenges and can make further elaboration difficult. As such, HTS hits may not offer the most ideal starting point for lead optimisation. These challenges drive the difficulty and cost of developing CNS drugs, culminating in the high failure rates of CNS drug discovery programs (Gribkoff and Kaczmarek, 2017). Therefore, incorporating alternative methods of identifying lead compounds that can accommodate the unique design challenges inherent in CNS drug discovery may ameliorate the high failure rates of CNS campaigns.

### 1.3 Fragment-based drug design

Fragment-based drug discovery (FBDD) is a method that utilises smaller, less complex, and non-specific structures known as “fragments.” These molecules can be thought of as building blocks to obtain a more customised, target-specific molecule. Fragments possess a specified degree of complexity as a proxy for the number of ligand–target binding interactions and are generally structurally guided by the “rule-of-three” (Ro3) (Table 1). (Congreve et al., 2003) Fragments obeying these criteria efficiently probe the structural morphology of a biological target, allowing key binding interactions to be deduced, which are in turn utilised to design a lead. More recent guidelines have fine-tuned the optimum molecular parameters which describe ideal fragments (Table 1). (Keserű et al., 2016) As a consequence of the lower complexity of the fragment-like molecules, their binding affinities are typically in the millimolar range, thereby necessitating biophysical screening techniques with a higher sensitivity than traditional methods. A chemically diverse fragment library of a few thousand, lower-complexity fragments can probe chemical space just as efficiently, if not more, than traditional large screening libraries whilst providing the same chemical space coverage. Furthermore, the smaller size of these FBDD-sourced leads will generally lead to higher ligand efficiency structures (which is a measure of how important each atom in a molecule is in establishing a strong binding interaction with the target). This may provide a means of resolving the issues of underperforming parameters during lead optimisation (Abad-Zapatero and Metz, 2005).

**TABLE 1:** Various parameters for an ideal fragment.

Rule-of-three (Ro3)	Parameters
Original	MW ≤ 300 Da; cLog*P* ≤ 3; HBD ≤ 3 HBA ≤ 3
Updated	150 ≤ MW ≤ 230 Da; 9–16 non hydrogen atoms; cLog*P* ≤ 2

#### 1.3.1 Fragment-based screening methods

FBDD programs utilise several biophysical methods for hit validation as a part of their screening process. Commonly, these techniques are used in tandem to verify the validity of a fragment hit. These screening methods have been reviewed in detail (Kirsch et al., 2019; Li, 2020; Togre et al., 2022); this section therefore serves as a brief summary of techniques that have been prominent in recent CNS drug discovery programs highlighted in this review.

Nuclear magnetic resonance (NMR) is an essential technique in FBDD to identify target binders, especially with weak interactions between the ligand and target, in a non-destructive manner. NMR screening can be categorised into two separate techniques: target-based and ligand-based screening. Target-based NMR utilises an isotopically labelled protein in two-dimensional (2D) experiments to identify binding mechanisms of the ligand with the protein (Singh et al., 2018). It is capable of determining if a ligand binds, and, if it does, where it binds by observing the chemical shifts experienced by the protein. This does, however, require high concentrations (>50 μM) of isotopically labelled proteins to produce examinable spectra (Stark and Powers, 2011). Comparatively, ligand-based methods rely on the change in NMR parameters between the proteins bound and ligand-free state in one-dimensional (1D) data (Di Carluccio et al., 2021). This technique does not observe changes in chemical shift in the protein but in the ligands instead; isotopic labelling of the protein is thus not required. Therefore, ligand-based NMR is ideal for proteins that can be difficult to express as it allows for the protein's concentration to be as low as 10 μM (Stark and Powers, 2011). Although this method can determine ligand binding, it is unable to deduce specific binding sites of the ligand. Furthermore, it has a higher chance of producing false negatives for medium-to high-binding affinity ligands with slow binding kinetics (Di Carluccio et al., 2021).

X-ray crystallography is a powerful tool that provides high-resolution structural information about the binding modality of the fragment to the protein. This binding modality can be determined regardless of the binding affinity of the fragment or the size of the protein (Hartshorn et al., 2005). This method is generally utilised in tandem with others as it does not provide a quantitative value for comparison, is limited by its low-medium throughput due to the time and effort in solving ligand-binding, and heavily relies on the solubility of the fragment and protein. Furthermore, this method is also limited to crystallisable proteins, which can make it challenging for targets such as membrane-bound proteins. Alternatively, cryogenic electron microscopy (cryo-EM) has become an increasingly popular technique for reconstructing the 3D structure of a molecule with the protein as protein crystals are not necessary (Callaway, 2020). However, for cases where experimental 3D structures of the protein are not available, predicted protein structures, using programs such as AlphaFold, can be generated for virtual screening purposes (Shi et al., 2023).

Surface plasmon resonance (SPR) is both a rapid and cost-effective method, utilising a high-throughput strategy to identify hits by measuring the kinetics of protein–ligand interactions (Navratilova and Hopkins, 2010). This screening method can be more advantageous than other current biophysical screening techniques, particularly due to its 10- to 100-fold lower protein consumption. Like ligand-based NMR, SPR can only identify whether a ligand will bind; it cannot verify the binding location. Furthermore, as fragments are typically tested in cocktails, SPR is unable to discriminate between structures that bind and those that do not. As such, a subsequent study is required with the individual fragments from cocktail mixtures to identify hit fragments (Farid E. Ahmed et al., 2010).

There are also a variety of auxiliary techniques that are used in FBDD campaigns. Thermal shift assays (TSA) are commonly used to measure the thermal stability of a purified recombinant protein or an isolated protein domain by measuring the change in the protein’s melting temperature when bound with a molecule (Jafari et al., 2014). Alternatively, cellular TSA (CETSA) can be performed if the whole cell or the cell lysates are available. Mass spectrometry (MS) is a rapid, autonomous, and highly sensitive method for identifying the binding of a fragment to a protein. The mass of the ligand–protein adduct can be observed to indicate successful binding (Dueñas et al., 2022). More recently, high-throughput affinity selection MS (HT-ASMS) introduced a cost-effective evaluation of large libraries by separating non-bound ligands from the protein through affinity enrichment or size exclusion chromatography. The bounded ligands are then be separated from the target protein, and their masses are accurately measured using MS. Liquid chromatographic-MS (LC-MS) can detect a reaction product within a mixture, requiring no substrate modifications, and is a label-free technique. In order to produce quantitative data, trypsin digestion is generally conducted to cleave proteins down into peptide fragments between 700 and 1,500 Da to ensure a readable mass (Laskay et al., 2013). By looking for the particular residue adduct, bound fragments could be distinguished from unbound. However, MS unfortunately limits the size of the protein due to the upper limit of the mass resolution (Chan et al., 2017). The protein sample must also be pure, as heterogeneity can increase the number of *m/z* signals in the spectra, resulting in increased complexity during analysis. Furthermore, MS and biochemical assays are also incompatible due to poor assay reproducibility and ion suppression from the high concentrations of salt, detergents, and buffering agents (Dueñas et al., 2022).

Unfortunately, there are difficulties faced by an FBDD campaign during its screening process. The fragments’ small size can limit the number of protein interactions, resulting in weak binding affinity (Shraga et al., 2021). Furthermore, biophysical screening requires higher concentrations of the fragments due to their weak binding, which can be a challenge for those with poor solubility. To circumvent this, the use of fragments with electrophilic moieties known as covalent fragments has started to emerge. These fragments allow covalent binding with the protein, alleviating the need for higher concentrations of fragments, as these interactions typically enhance a fragment's activity. This technique is based on disulfide tethering, which allows for a reversible disulfide bond exchange between a molecule and the target protein containing a cysteine residue near the binding site as they are the most nucleophilic residues (Kathman and Statsyuk, 2016). However, this is not limited to the use of disulfides, as it is possible with other groups such as acrylamide and vinylsulfone, which the fragment may already contain. It can also be site-directing by introducing a fabricated cystine on the target protein that does not have one available. The screening process of these covalent fragments is typically done on LC-MS, which allows mixtures of fragments to be evaluated. Once covalent hits are identified, they can be reverted to their non-covalent counterpart to be assessed at the same binding site. However, it has become increasingly popular to use these as a starting point for covalent inhibitors. The benefits of these fragments is their increased potency, improved selectivity, and prolonged duration of activity and exposure. Regardless, once a fragment hit is identified, a subsequent elaboration process will develop these building blocks into a drug-like lead.

#### 1.3.2 Fragment elaboration

**FIGURE 1:**
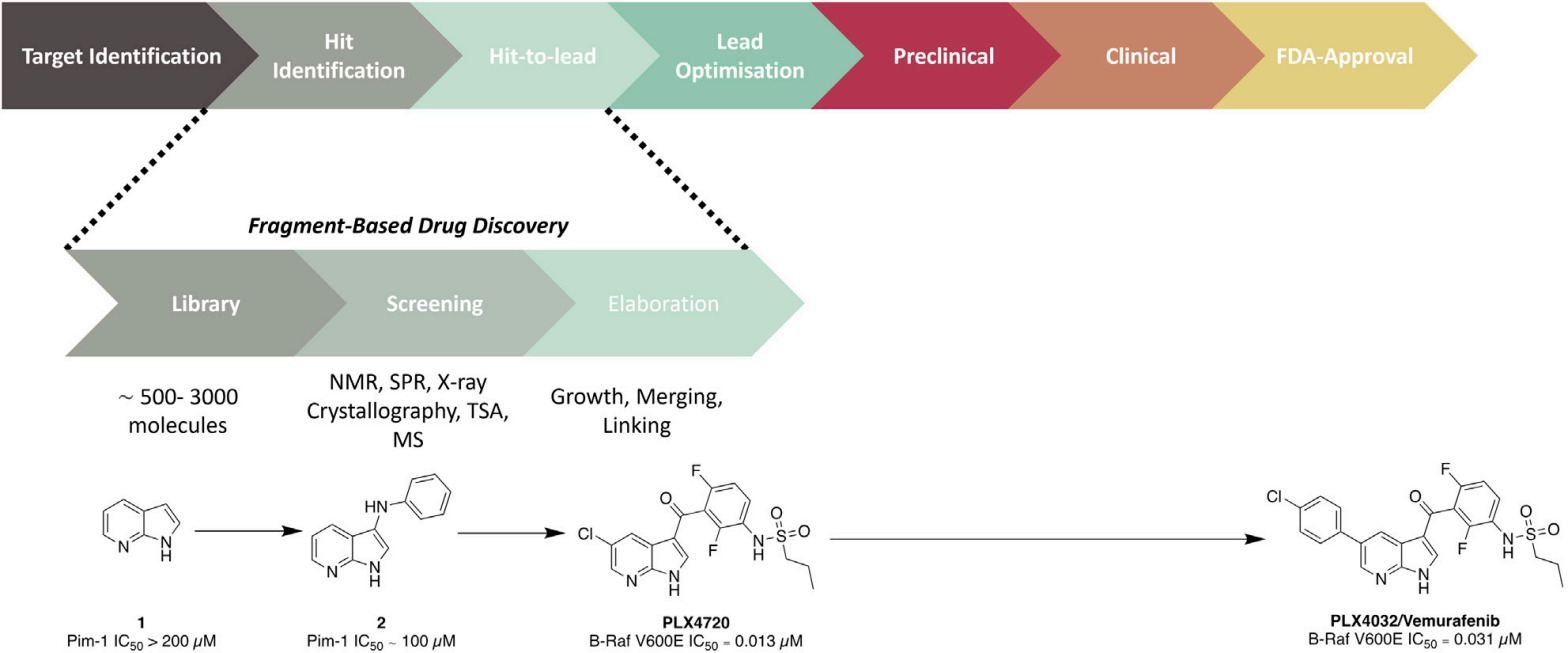
Overview of an FBDD campaign. The development of the late-stage melanoma treatment *Vemurafenib*, which took only 6 years from the start of the project to approval (Shi et al., 2023), is provided as an illustrative example.

Fragment hits generally possess weak millimolar binding affinities and little to no potency effects on the target. Therefore, guided structural modifications are required to advance these fragments from a hit to a lead compound. The aim in this procedure is to improve the binding affinity/activity of the structure whilst transitioning to a drug-like molecule. This process can be achieved through either fragment growth, linking, or merging. Fragment growth is the most common method, where a hit is taken through a structure–activity relationship (SAR) study to add structural motifs. This is possible even without binding data; however, it is crucial that some form of biochemical or biophysical assay is conducted to direct the SAR study. Fragment linking involves the joining of two or more fragments which recess in different subdomains of the protein. This process is generally undertaken for targets with large binding sites so that there are clear identifications of fragments binding in different regions of the pocket. This method relies on data provided through NMR or x-ray crystallography to understand the binding mode of individual fragments and the combined compounds. However, one of the challenges with this strategy is developing a linker that connects fragments that have no negative effect on the overall activity of the molecule. Finally, fragment merging, which also relies on understanding binding modes, is an alternative strategy to identify more drug-like core structures or molecules. This method aims to merge hit fragments that occupy overlapping space in the binding site to generate an optimised pharmacophore. With the successful elaboration of a fragment hit to a lead molecule, further optimisation is undertaken to advance a lead along the drug discovery pipeline. To date, there have been six FBDD derived drugs that are FDA-approved: *Vemurafenib* (Figure 1) (Bollag et al., 2012), *Erdafitnib* (Murray et al., 2019), *Sotorasib* (Lanman et al., 2020), *Venetoclax* (Souers et al., 2013), *Pexidartinib* (Benner et al., 2020), and *Asciminib* (Schoepfer et al., 2018).

## 2 Applications and advances of CNS studies with FBDD

This section highlights CNS drug discovery programs that have utilised FBDD since 2015, when the last review in this field was published by Wasko et al. (2015). Each of the studies will be categorised by indications and the specific targets of interest. For each study, we explore the various screening techniques and libraries used to obtain a hit and how this hit was elaborated to an improved lead. Where possible, we also compare the FBDD-derived molecules to those identified *via* other methodologies.

### 2.1 Alzheimer’s disease

Alzheimer’s disease (AD) is commonly characterised by dementia, which includes cognitive deterioration, memory loss, and personality and behavioural abnormalities. Although there are treatments that temporarily alleviate symptoms, there are no disease-modifying treatments currently available. The number of people living with dementia (60%–70% attributed to AD) globally has reached over 55 million (Greenblat, 2023), which is more than double the 20.3 million reported in 1990 (Li et al., 2022).

#### 2.1.1 Apolipoprotein E (apoE)

One of the theoretical causes of AD is the accumulation and deposition of amyloid-beta (Aβ) peptides in the brain. This increase of Aβ peptides is most commonly associated with the gene, *apolipoprotein E* (apoE). ApoE has roles in brain homeostasis, and there is emerging evidence that it mediates one of the many pathways for the clearance of Aβ, although the precise mechanisms are currently unclear (Yamazaki et al., 2019). There are several isoforms of apoE which each possess varying degrees of efficiency in Aβ clearance, with apoE4 the slowest. ApoE4 also appears to promote Aβ aggregation due to its lower stability profile than other isoforms. As such, individuals possessing a pair of apoE4 alleles have the strongest genetic risk factor for AD (Yamazaki et al., 2019).

**FIGURE 2:**
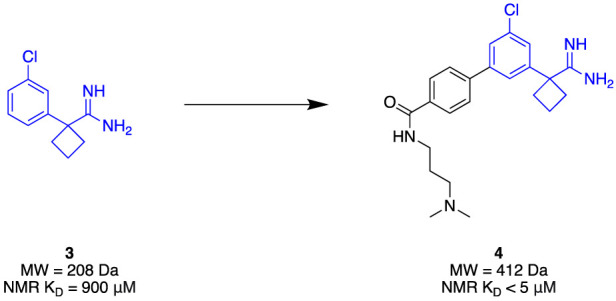
APOE4 Fragment hit identification to the lead molecule.

Given its clinical relevance, apoE4 has become a therapeutic target of interest. It has been theorised that increasing the stability of apoE4 will improve Aβ clearance as well as minimise aggregation (Petros et al., 2019). The N-terminal domain of apoE4 was seen as a suitable target due to its small, stable nature. Due to its small binding site and the limited number of molecules associated with apoE4, an FBDD strategy is well-suited to efficiently explore the target for novel chemotypes. Petros et al. (2019) utilised a combination of ^13^C-HSQC fragment screening, SPR, and TSA to identify a suitable apoE4 stabiliser. A library of 4068 fragments was first screened using ^13^C-HSQC NMR against the N-terminal domain of apoE4 that was ^13^C-labelled. Each fragment complied with Ro3, and they were assayed as a cocktail mixture of 12. A genuine hit was considered to cause a minimum shift of 0.05 ppm and 0.5 ppm in the proton and carbon spectra, respectively. The hits then underwent NMR titration to determine binding affinity, which identified amidine **3** (Figure 2) as the highest affinity binder (K_*D*_ = 900 µM) at the apoE4 N-terminal domain. Amidine **3** was further subjected to SPR to obtain its binding affinity of K_D_ = 205 μM at the N-terminal domain of apoE4. In addition, the binding affinity with the full length of apoE4 from mammalian cell expression, and the full length apoE3 (isoform) resulting in K_D_ = 233 μM and K_D_ = 890 µM, respectively, represented good selectivity towards apoE4. Furthermore, stabilisation of the protein was observed with the addition of 5 nM of the fragment, improving thermal stability by approximately 4°C in comparison with the lone protein. With excellent preliminary results, fragment growth was conducted to transform the fragment into lead **4** that exhibited NMR-derived affinity of K_D_ < 5 µM whilst retaining the same thermal stability amount through TSA of initial fragment **3** at a five-fold lower concentration. The conclusion of this study demonstrated a successful identification of a stabiliser for apoE4 through a streamlined elaboration that was enabled through a comprehensive FBDD campaign.

#### 2.1.2 Lipoprotein-associated phospholipase A2 (Lp-PLA2)

**FIGURE 3:**
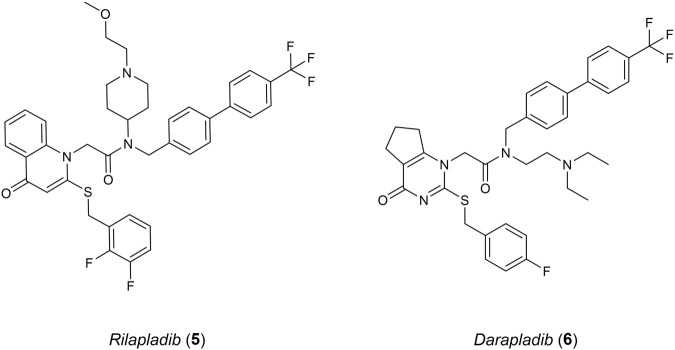
Known Lp-PLA_2_ inhibitors.

Studies surrounding lipoprotein-associated phospholipase A2 (Lp-PLA_2_) have linked an increased expression of this protein in humans to AD and atherosclerosis (Woolford et al., 2016). LP-PLA_2_ belongs to a group of the phospholipase A2 superfamily, cleaving *sn*-2 ester bonds of glycerophospholipids such as platelet activating factor (PAF). Elevated levels of Lp-PLA_2_ cause oxidised modifications of phospholipids into various pro-inflammatory stimuli which include lysophosphatidylcholine and oxidised non-esterified fatty acids. Oxidative stress and inflammation occur as a result. Lp-PLA_2_ has become a suitable inflammatory biomarker for cardiovascular disease (CVD) and heart disease (Doody et al., 2015). However, understanding the relationship between Lp-PLA_2_ and AD is still difficult, as risk factors such as CVD could have an influence in its progression. *Rilapladib* (**5**), a known Lp-PLA_2_ inhibitor (Figure 3), was able to provide initial evidence supporting Lp-PLA_2_ as a treatment for AD (Maher-Edwards et al., 2015). Among the class for Lp-PLA_2_ inhibitors, *Darapladib* (**6**) has been the most promising, advancing into phase III clinical trials originally as a therapeutic for atherosclerosis but failing to meet efficacy requirements (Woolford et al., 2016).

**FIGURE 4:**
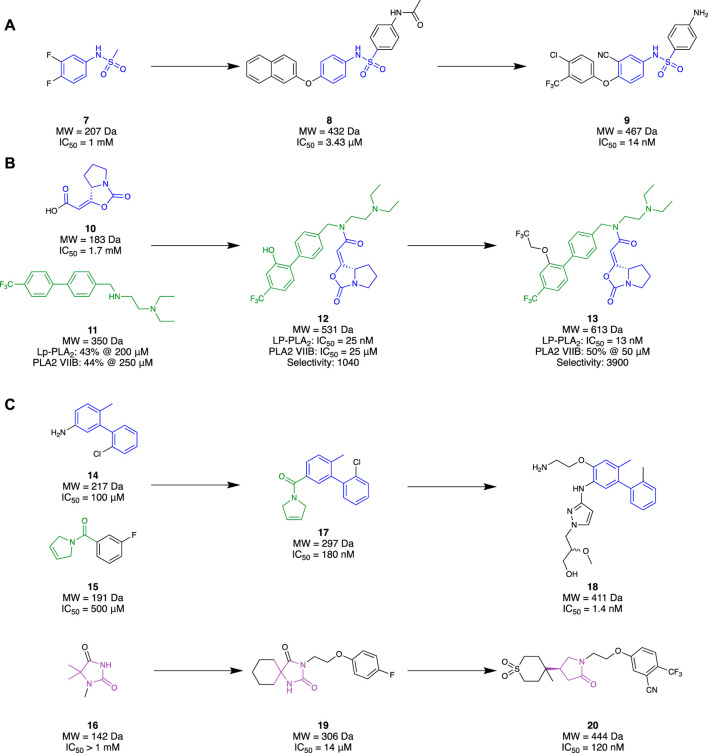
Lp-PLA_2_ fragment hit identification to the lead molecule. **(A)** Liu and co, **(B)**, Huang and co development of a covalent ligand, **(C)** GlaxoSmithKline.

Initially, **6** presented itself as a promising molecule to Chen et al. (2015), where its high potency prompted SAR studies to improve oral efficacy, allowing it to be repurposed as an AD therapeutic. Unfortunately, improving the oral bioavailability of these analogues proved to be challenging, mainly due to their already large molecular size and high lipophilicity. Liu et al. (2017) followed up this study with an FBDD campaign with the aim of identifying a novel scaffold that could improve the physicochemical properties of **6** whilst maintaining a comparable potency and selectivity profile. Virtual screening was initially conducted with a library of 500 in-house fragments utilising a co-crystal structure of a recombinant human Lp-PLA_2_ (rhLp-PLA_2_). This allowed for observations of each fragment’s occupancy in the binding pocket and led to fragment **7** (IC_50_ = 1 mM, LE = 0.30) that exhibited similar interactions to **6**. Utilising the SPECS database, molecules that contained the structural moiety of **7** underwent molecular docking studies using the co-crystal structure of the sulfonamide with rhLp-PLA_2_ to grow the fragment to fill the binding site. The 500 highest ranked compounds were filtered, prioritising those that had dissimilar binding modes to the original small fragment. This was followed by cluster analysis using a leader–follower method to obtain 100 structures. Finally, a PAF enzymatic assay was conducted with the remaining molecules, obtained either commercially or in-house, which identified potent structure **8** (IC_50_ = 3.43 µM) as a hit (Figure 4A). Structure **8** was subsequently elaborated, resulting in sulfonamide **9**. Compound **9** demonstrated good *in vivo* clearance in male rat hepatocytes (CL = 4.9 mL/min/kg), favourable AUC value (3.4 μg h/mL), and good oral bioavailability (*F* = 35.5%). Furthermore, it maintained inhibitory activity 24 h after oral administration, which is superior to **6** exhibiting inhibitory activity for only 8 h. With the success of this campaign, **9** is currently under further optimization, which illustrates that a successful fragment-based campaign allowed for an accelerated hit-to-lead transition.

Huang et al. (2020) also had success with an FBDD campaign which aimed to create covalent ligands as a drug for AD or biochemical tool for Lp-PLA_2_. Serine and cysteine residues are most often exploited to form covalent interactions with small molecules, given their nucleophilicity under physiological conditions. As serine (Ser273) was located deep within the binding pocket of Lp-PLA_2_, it was chosen as the ideal residue to investigate through a fragment-based approach. Ideally, a small molecule that covalently binds to the Ser273 residue would be grown into a drug-like molecule, without sacrificing binding efficiency. Through a deconvolution study of three known inhibitors that bound to Lp-PLA_2_, **10** was chosen as the covalent binding moiety as it possessed good selectivity for Lp-PLA_2_ over other serine hydrolases. Elaboration of **10** began with hybridisation with **11** to form **12**, a molecule strikingly similar to the previous gold standard, **6**. Both individual fragments **10** and **11** showed millimolar potency with no selectivity towards Lp-PLA_2_ over PLABVIIB. However, their hybrid, **12**, exhibited a 68000-fold improvement in IC_50_ (IC_50_ = 25 nM) and 1000-fold improvement in selectivity for Lp-PLA_2_ (Figure 4B). Comparison of the co-crystal structures of hybrid **12** and **10** illustrated that the binding conformation of the small fragment was highly conserved. Similarly, the comparison of the co-crystal structure of **12** with **6** illustrated the conserved binding pose of the biphenyl group, recessed in a hydrophobic sub-pocket. Therefore, minimal changes were made to enhance the synergistic behaviour of the fragments, only filling the unoccupied sub-pockets regions to obtain their lead molecule **13**. The addition of a linear 1,1,1-trifluoroethyoxyl group on the *meta*-position of the second biphenyl ring exhibited a two-fold increase in the inhibitory response (IC_50_ = 13 nM) and a four-fold increase in selectivity for Lp-PLA_2_ compared to hybrid **12**. Furthermore, it exhibited exceptional selectivity over a wide selection of serine hydrolases and a three-fold improvement in selectivity compared to **6** for Lp-PLA_2_. Such exemplary results illustrate that a covalent fragment-based campaign can be a promising alternative to conventional approaches where directly probing an inhibitor to introduce covalent properties may not be feasible.

Researchers from GlaxoSmithKline (GSK) also chose to explore novel chemical space for Lp-PLA_2_ utilising an FBDD strategy (A. J. A. Woolford et al., 2016). Unlike the previous research discussed, GSK utilised a commercialised fragment screening platform from Astex Pharmaceuticals against Lp-PLA_2_. This platform utilised a combination of TSA, NMR, and x-ray crystallography experiments to identify a desirable fragment hit. An in-house crystal system was also developed to facilitate the fragment soaking and data collection. A library of 1,360 small fragments was screened in cocktail mixtures of four, where a subset of 150 fragments was subjected to x-ray crystallography. The fragment hits that were identified from TSA or ligand-detected NMR (LD-NMR) were only pursued if they could be validated *via* x-ray crystallography. This resulted in a total of 34 fragments, with an additional 16 identified from a substructure search around the x-ray-validated fragments. Interestingly, superimposing the crystal structures of a select group of five fragment hits illustrated a collective binding surface comparable to **6**. However, a collection of bis-aryl fragments such as **14** (IC_50_ = ∼100 μM, LE = ∼0.36) was found to bind in a novel binding site. Superimposing the binding mode of one of the other five fragment groups, an amide **15** (IC_50_ = ∼500 µM) with **14** saw similar binding behaviours in the pocket. Both fragments harboured interactions in different areas of the pocket, leading to the design of their hybrid fragment **17** (IC_50_ = 180 nM, LE = 0.44). Subsequent growth of fragment **17** resulted in compound **18** (IC_50_ = 1.4 nM) with over 1000-fold selectivity over PLA2-VIIB. Although it possessed improved physicochemical properties (cLog*P* = 3.4, aqueous solubility = 302 μM) compared to **6** (cLog*P* = 8.2, solubility = 8 μM), it did not possess a suitable PK profile (Cl_int_ = 10.2 mL/hr/mg) required for once-daily dosing in humans, highlighting a major challenge in drug development and subsequently putting a halt to this study (Figure 4C).

In conjunction with the previously discussed study, GSK also pursued a selection of fragments with the aim of designing an inhibitor capable of matching the activity of **6** in a plasma assay (Woolford et al., 2016). They hypothesised that a lead molecule might not need to possess picomolar activity if the discrepancy between Lp-PLA_2_ biochemical and plasma assays could be reduced. Such an approach could potentially lead to a reduction in MW, resulting in a more favourable level of lipophilicity than **5**. Sharing the same fragment-based screening output as above, 50 fragments were prepared for x-ray crystallography to observe their binding at the target. This resulted in three distinct fragment hits that occupied different regions of the binding pocket. Specifically, a hydantoin fragment **16** (IC_50_ > 1 mM, cLog*P* = 0.03) represented a good starting point due to its small size and convenient synthetic accessibility for exploring the binding pocket through different growth vectors (Figure 4C). Hydantoin **16** occupied the oxyanion hole with two hydrogen bonds to the NH of the amide junction between Leu153 and Phe274. However, due to its significantly reduced activity than the other hit fragments, a virtual screening strategy was implemented to combine motifs of hydantoin **16** with the other hit fragments that occupy adjacent sites of the binding pocket with notable activity. Approximately 16,000 hydantoin fragments commercially obtained or prepared in-house were screened against an in-house Lp-PLA_2_ crystal structure utilising the Astex proprietary version of Gold software to grow the fragment, resulting in structure **19** (IC_50_ = 14 μM, LE = 0.30) as the favourable hit molecule. Further structural elaboration of **19**, which included the replacement of the hydantoin with a γ-lactam, resulted in lactam **20** (IC_50_ = 0.12 µM, LE = 0.35, and cLog*P* = 0.75) —a low MW, potent inhibitor with favourable lipophilicity which exhibited selectivity against PLA2-VIIB and no significant inhibition of CYP enzymes. The design of lactam **20** demonstrates an alternative potent and selective inhibitor that possesses favourable PK properties and low MW.

These studies surrounding Lp-PLA_2_ present great examples of the benefits and shortcomings of FBDD. Previous rational drug design has seen challenges in obtaining a potent molecule that retains desirable lipophilicity when working with larger drug-like molecules. However, the fragment-based strategy allows for the constructive identification of fragment binders possessing desirable qualities, followed by the coherent optimisation of the molecule to improve its potency whilst retaining desirable parameters.

#### 2.1.3 Notum

The wingless-related integration site (Wnt) signalling pathway regulates important factors associated with cell fate determination, migration, polarity, and neural patterning. However, the carboxylesterase Notum has been implicated in the suppression of Wnt signalling through the deacylation of essential palmitoleate groups on Wnt proteins. As Wnt signalling contributes to healthy brain function and synaptic plasticity, its suppression has been implicated in AD (Palomer et al., 2019). Wnt signalling has been considered a challenging target for the medicinal chemist as many targets are not considered “classically druggable” (Mahy et al., 2020b).

**FIGURE 5:**
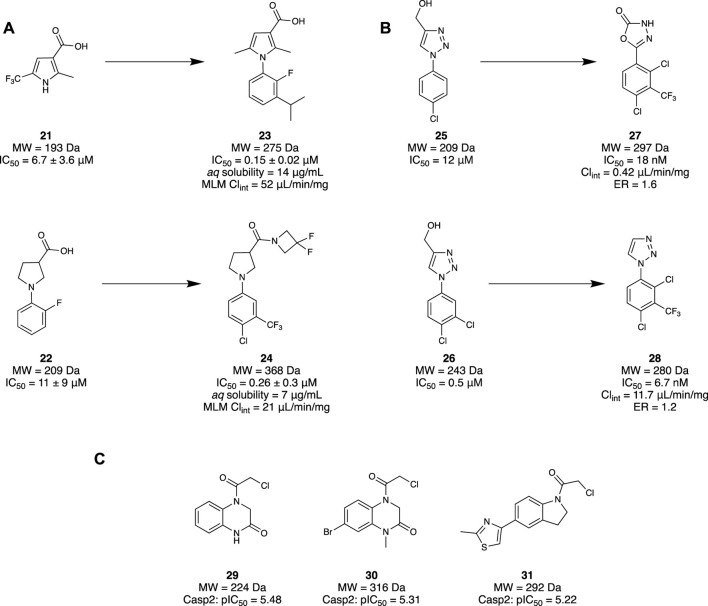
**(A)** Examples of the set of pyrrole-3-carboxylic acid **21** and pyrrolidine-3-carboxylic acid **22** structural motifs. **(B)** Identification of two fragment hits for Notum and their subsequent lead molecule for Notum. **(C)** Final fragment hits with potential to become Casp2 inhibitors.

Mahy et al. (2020a) utilised an FBDD approach to investigate a novel class of Notum inhibitors to gain new insight into Wnt signalling. A library of 4,350 fragments sourced from the Enamine Carboxylic Acids Fragment library was used due to its novelty and diversity. A subsequent collection of 250 fragments was made through a selection process based on molecular properties, structural parameters, physicochemical property space, and structural diversity. Through a Notum 8-octanoyloxypyrene-1,3,6-trisulfonate (OPTS) biochemical assay which measured suppressed fluorescence with binding to Notum and crystallography study, 14 fragments were selected. These were found to bind in the palmitoleate pocket of Notum, revealing interactions with the Phe268 and Trp128—favourable for inhibitory activity. A set of pyrrole-3-carboxylic acid **21** and pyrrolidine-3-carboxylic acid **22** motifs were subsequently selected as their leads, whilst the remaining hits were used to help guide the SAR investigation (Figure 5A). These two lead motifs were grown to better fill the binding pocket, revealing four lead-like molecules, such as **23** and **24**, with low to sub-micromolar potency. Unfortunately, even though these fragment-derived molecules were able to inhibit Notum, they remained inferior to other lead molecules identified through traditional screening strategies.

Following this study, Mahy et al. (2020a) then published another investigation revealing a class of potent inhibitors of Notum. An x-ray crystallography fragment screening method was performed at the XChem platform of Diamond Light Source using 768 compounds from the DSPL fragment library. This library of 768 fragments was assessed in a crystallographic fragment screen using the XChem platform to seek fragments that bind in the palmitoleate pocket. A diverse group of 60 fragments were identified, including **25** and **26**. Molecule **25** demonstrated an exemplary binding mode, with modest potency (IC_50_ = 11.5 μM). Hence, subsequent efforts were made to elaborate both ring systems of **25**, wherein the triazole ring was hypothesized to participate in crucial H-bonding interactions. As a result, the triazole ring was replaced with an oxadiazole to improve H bonding capabilities, yielding compound **27**. Compound **27** maintained a fragment-like size and a nanomolar potency (IC_50_ = 18 nM), good metabolic stability (Cl_int_ = 0.42 μL/min/mg), and high cell permeability with a low efflux ratio (ER = 1.6) (Figure 5B).

A subsequent study from Willis and colleagues examined the same compound library using the same screening method, this time examining compound **26** as the lead compound. Fragments **25** and **26** differ only by a meta-chloro group on the phenyl ring. However, fragment **26** possessed slightly better potency (IC_50_ = 0.5 µM), good aqueous solubility (100 μg/mL), moderate stability in mouse liver microsomes (MLM) protein (Cl_int_ = 88 μL/min/mg), and excellent cell permeability (ER = 1.0), consistent with a lead-like molecule. Unsurprisingly, both compounds bind in the same pocket and conformation. Efforts to optimise compound **26** aimed to maintain the vital triazole interactions, instead focusing on functionalising the phenol ring in the southern portion of the molecule. Minimal elaboration was required to access their lead molecule **28** with nanomolar inhibitory activity (IC_50_ = 6.7 nM), involving the removal of the alcohol group on the triazole and an additional CF_3_ group on the phenyl. X-ray crystallography of **28** demonstrated retention of the H-bonding interaction of the triazole with Trp128. Furthermore, with a trisubstituted phenyl ring, the palmitoleate pocket was nearly completely occupied. In addition to the high potency, the physicochemical properties were consistent with a drug-like molecule, displaying good aqueous solubility (solubility = 240 µM), MLM stability (Cl_int_ = 11.7 μL/min/mg), and high permeability (ER = 1.2) (Figure 5B). *In vitro* studies of **28** demonstrated restored Wnt signalling in the presence of Notum, and subsequent *in vivo* results illustrated good oral bioavailability and brain penetration as drug concentrations in the brain and plasma were similar. The collective result of these studies demonstrates the identification of a novel class of inhibitor for Notum through a successful FBDD campaign, and a new chemical probe to further support the implications of Notum and AD.

#### 2.1.4 Caspase-2

Caspase-2 (Casp2) is a cysteine protease that has generally been targeted using peptidic and peptidomimetic ligands. Casp2 cleaves neuronal scaffold-protein tau, leading to neurofibrillary tangles resulting in reversible synaptic dysfunction, thus making it a potential target for AD *via* synaptic function restoration (Vigneswara and Ahmed, 2020). Focus has recently been drawn to developing inhibitors with covalent reversible and irreversible binding modes (Cuellar et al., 2023). This form of binding is possible through electrophilic moieties and has demonstrated validity in both reversible and irreversible cysteine protease inhibitors.

Cuellar et al. (2023) have tried to develop a small-molecule inhibitor through a fragment scaffold which utilises an electrophilic screening strategy to overcome the CNS drug challenges faced by pentapeptides and peptidomimetics. These generally have several H-bond donors and a molecular weight that are incompatible with passive diffusion into the brain, and therefore suffer from poor brain bioavailability. Incorporating an α-chloroacetamide library from Enamine, 1,920 compounds were tested for the inhibitory effects on Casp2 and Casp3 due to their similar substrate recognition sequence using a fluorometric enzyme assay. α-chloroacetamides were chosen as there is precedent in the literature for these groups to undergo covalent bonding against cysteine proteases (Resnick et al., 2019; Gao et al., 2022). The screening of the compounds was conducted at two concentrations for both Casp2 (12.5 and 6.25 µM) and Casp3 (125 and 62.5 µM). A three-sigma rule (µ + 3 σ) was used to determine molecules that were at least three standard deviations (σ) greater in inhibition activity from the mean (µ) and class them as hits. The results from the 12.5 µM concentration screen provided better differentiation between the molecule and baseline, resulting in 64 hits that could yield an inhibition greater than 60% with Casp2. Five compounds stood out, with inhibition values ≥90%. An identical method was used to identify compounds with inhibitory activity at Casp3, resulting in 72 hits. However, significantly lower values were obtained when the experiment was repeated at 12.5 µM to align with values obtained for Casp2. Comparing the data and calculations of pIC_50_ values to determine selectivity profiles, a select group of compounds were found to have the highest affinities and single digit micromolar activity. To investigate the covalent binding of the fragments to Casp2, mass spectroscopy peptide sequencing using LC-MS was conducted for a target engagement study. To illustrate the irreversibility of these fragments to the active site of Cys320, the fragments and other reference molecules were incubated with Casp2. Trypsin digestion of the protein/fragment adduct was performed, ensuring observation of the protein fragment MFFIQA**C**_**320**_R, as this contained the active site Cys320. The results confirmed the covalent irreversibility binding of all the electrophilic fragments to Cys320. Hit validation was then conducted with three of the promising hits, fragments **29**–**31** (Figure 5C), and resubjecting them to another fluorometric enzyme assay. All hits demonstrated affinity to Casp2 in a single-digit micromolar range with no significant Casp3 inhibition. Overall, it was demonstrated that a selection of electrophilic fragments with promising affinity and selectivity for Casp2 could create a diverse fragment scaffold. As they all observed low micromolar inhibitory concentrations, they could provide a great foundation for developing a selective small-molecule Casp2 inhibitor.

#### 2.1.5 Sortilin

**FIGURE 6:**
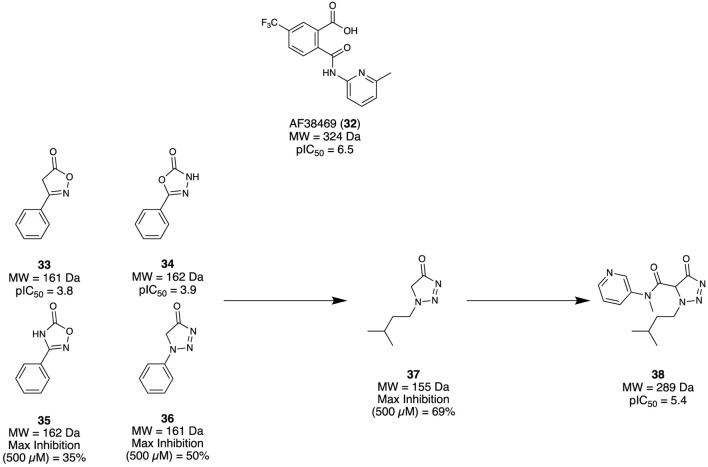
Fragment hit identification to the lead molecule for sortilin.

Sortilin is a membrane protein that mediates several physiological functions through trafficking and signalling with different protein partners. It has become a promising therapeutic target due to its implication with several disease states, including AD. Andersen et al. (2017) aimed to identify a modulator for Sortilin as a possible therapeutic intervention. Previously, Schrøder et al. (2014) had reported an orally bioavailable small molecule AF38469 (**32**) developed through HTS screening; however, it exhibited poor CNS exposure, making it unsuitable as a tool for *in vivo* studies. As the carboxylic acid of **32** was deemed responsible for the low CNS exposure, an FBDD campaign exploring suitable chemotype replacements for this moiety was undertaken. Utilising the Lundbeck Fragment Library, 1,600 compounds were screened using a previously reported neurotensin binding scintillation proximity assay (Schrøder et al., 2014). Among the four fragments identified, **33** and **34** both demonstrated adequate potency to produce a concentration response curve and calculative IC_50_, while **35** and **36** could not but still demonstrated a reproducible specific inhibition at the highest assay concentration (Figure 6). Taking these initial hits (**33–36**), the first round of modifications led to fragment **37** with max. inhibition of 69%. Subsequent elaborations aimed to improve potency by expanding the fragment to occupy similar regions demonstrated by **32**, which afforded the lead compound **38** (pIC_50_ = 5.4, cLog*P* = 2.2, MDCK cell line permeability 2.9 cm/s × 10^−6^). Overall, a novel cell-permeable sortilin inhibitor was successfully identified with good potency. It was found that further elaboration of **38** could yield a better optimised interaction with sortilin, resulting in improved potency, permeability, and possible CNS exposure.

### 2.2 Parkinson’s disease

Parkinson’s disease (Parkinson’s) is a brain disorder diagnosed by uncontrollable movements that manifest symptomatically as tremors, shaking, stiffness, and slow movements as well as a loss of unconscious movements, sense of smell, and fatigue. Typical homeostatic function normally mediates the production of dopamine, which acts as a neurotransmitter between the body and brain to regulate body movements and emotions. In cases of Parkinson’s, the basal ganglia become impaired and/or die, resulting in a reduced production of dopamine. Dopaminergic loss is one of the pathological indicators of Parkinson’s. Monoamine oxidase-B (MAO-B) is an enzyme associated with Parkinson’s and has been classed as a pharmacological target for its treatment, as its inhibition increases dopamine levels (Tan et al., 2022). There have been a variety of inhibitors developed for MAO-B, such as *tranylcypromine* and *phenelzine—*two nonselective, irreversible inhibitors. These can, however, lead to tyramine build-up due to the consumption of tyramine-rich foods, resulting in a hypertensive crisis called the “cheese effect.”

**FIGURE 7:**
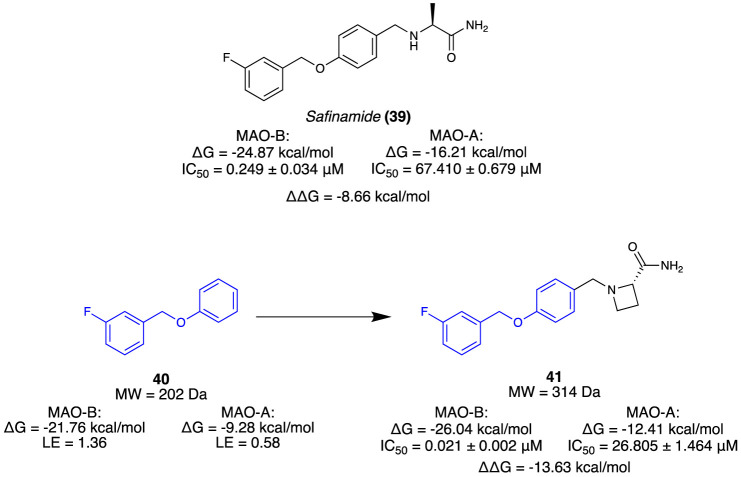
Known inhibitor of MAO-B. Fragment selection through steric stress to lead molecule for MAO-B.

Jin et al. (2020) aimed to isolate a selective, reversible inhibitor for MAO-B by incorporating a novel computational strategy called steric clash-induced binding allostery (SCIBA). MAO-A and MAO-B share a highly conserved protein sequence, except for a pair of residues (MAO-B has Ile199 in place of Phe208 and MAO-A has the inverse). The residue variation was considered the reason for the favourable binding mode of the linear-type *Safinamide* (**39**) in MAO-B whilst being unsuited for the curved binding site of MAO-A (Figure 7). SCIBA was used to identify fragments with steric clashes with MAO-A proteins and select them as fragments that possess favourable PK properties at MAO-B. Borne from the west half of **39**, fragment **40** observed steric clashing with the sub-pocket of MAO-A due to the residue difference. It was thought to result in a conformational change of **39**, weakening the binding affinity. A deconvolution study of **39** was conducted, calculating the binding free energy (ΔG) and ligand efficiency (LE) of each fragment. A fragment library was assembled using a database of FDA-approved drugs, and fragments were sorted based on the ΔG value towards MAO-B. Using **40** as the hit fragment, only minimal growth was required to fill the unoccupied sub-pocket where falvin adenin dinucleotide (FAD) cofactor resided, resulting in a series of *(S)*-2-(benzylamino)propenamides (Figure 7). Subsequent *in vitro* results indicated that all the derivatives had no inhibitory activity at MAO-A. Compound **41** (IC_50_ = 0.021 µM) was selected as the lead as it exhibited high inhibitory activity at MAO-B and no activity at MAO-A. Reversibility as 100% recovery of MAO-B was observed after 24 h, whilst also exhibiting similar increases of dopamine levels compared to **39** (Figure 7). Overall, Jin et al. (2020) demonstrate a novel fragment approach for identifying favourable motifs of a target by observing steric clashing at an off-target site, resulting in a successful hit campaign and a potential lead compound for the treatment of Parkinson’s through minimal optimisation.

### 2.3 Schizophrenia

Schizophrenia is a chronic, debilitating disorder that impairs the psyche and motor function of the brain. Schizophrenia is characterised by positive, negative, and cognitive symptoms that manifest as delusions and hallucinations, amotivation and apathy, and deficits in memory and learning ability (McCutcheon et al., 2020). The current understanding of its pathology is that there is no singular target that can be drugged to cure the disease. Current therapies focus on antagonism of the dopamine 2 (D2) receptor to treat positive symptoms, whilst there are little to no effective therapies for negative symptoms or cognitive impairments (McCutcheon et al., 2020).

#### 2.3.1 Phosphodiesterase 10A

Phosphodiesterases (PDEs) are a family of enzymes that control the hydrolysis of signal messengers, cyclic adenosine monophosphate (cAMP), and cyclic guanosine monophosphate (cGMP). This family of enzymes regulates the concentration of cAMP and cGMP to maintain healthy signalling in the brain (Zagorska et al., 2018). Inhibition of the PDE isoform 10A (PDE10A) is considered effective in the treatment of a range of psychological, neurological, and movement disorders associated with schizophrenia (Zagorska et al., 2018; Amin et al., 2021).

**FIGURE 8:**
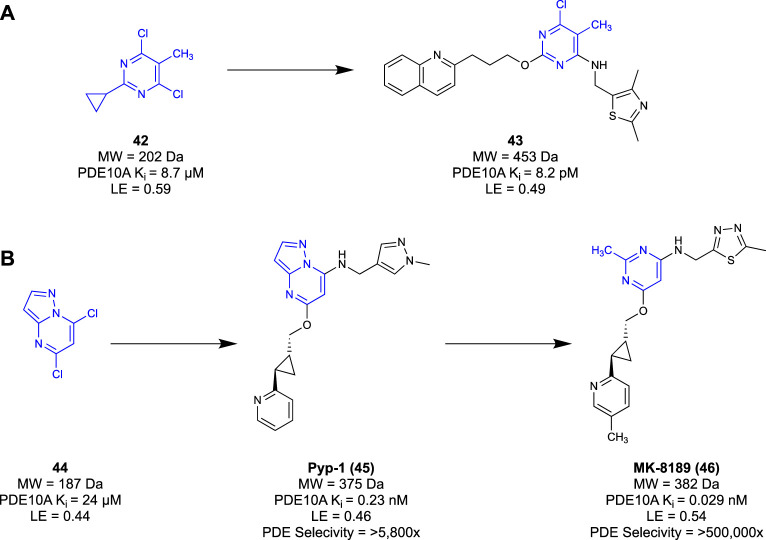
**(A)** Efforts by Shipe et al., while the lower panel highlights work from Raheem et al. and Layton et al. **(B)** Fragment to lead elaboration of MK-8189 from Merck.

Researchers from Merck discovered a novel chemotype of PDE10A antagonists through a dual fragment and conventional drug discovery campaign (Shipe et al., 2015). HTS was conducted using the proprietary Merck compound library, yielding hit and subsequent lead compounds that would ultimately suffer from poor physicochemical characteristics. Compounds from this series were used as PET tracers (Cox et al., 2015) or benchmark compounds for biochemical assays (Raheem et al., 2012) but were never utilised as candidates for further development. A subsequent fragment screen of the Merck fragment libraries against an *in vitro* PDE10A2 inhibition assay delivered a pyrimidine-based fragment hit **42**. The compounds developed from this fragment were synthesised in parallel, with SAR decisions informed by an *in silico* structure-based study. The pyrimidine fragment was elaborated into candidate **43**, showing an over 1-million-fold improvement in potency relative to the initial fragment hit **42** (Ki = 8.2 pM vs 8.7 μM, respectively). Unfortunately, like the series identified in HTS, this generation of compounds suffered low oral bioavailability and high clearance, amongst other issues, ceasing development. The next generation of antagonists was reported by Raheem et al. (2016) using the fragment hits discarded from the initial fragment screen. In an attempt to address the unfavourable PK profile that ceased development of candidate **42**, fragment core **44** was selected. This had a lower LE and potency, but it did not suffer from the clearance, bioavailability, and selectivity issues seen in the previous generation. The next-generation core was rapidly optimised, utilising data from the *in silico* study as well as knowledge gained from the initial fragment screen. This development led to compound PYP-1 (**45**), with similar potency to the previous generation of compounds but with less metabolic liabilities. A subsequent study by Layton et al. (2023) refined both the east and west wings of **45**, resulting in slight potency gains. They then re-examined the pyrazolopyrimidine core of the molecule to investigate nitrogen placement in the heterocyclic ring. Subsequent SAR studies concluded that a 2-methyl-pyridmidine core was the most favourable moiety. This research culminated in 2020, when MK-8189 (**46**) (Figure 8B), a lead compound borne of **45**, entered phase I clinical trials and was well-tolerated by patients (Layton et al., 2023).

**FIGURE 9:**
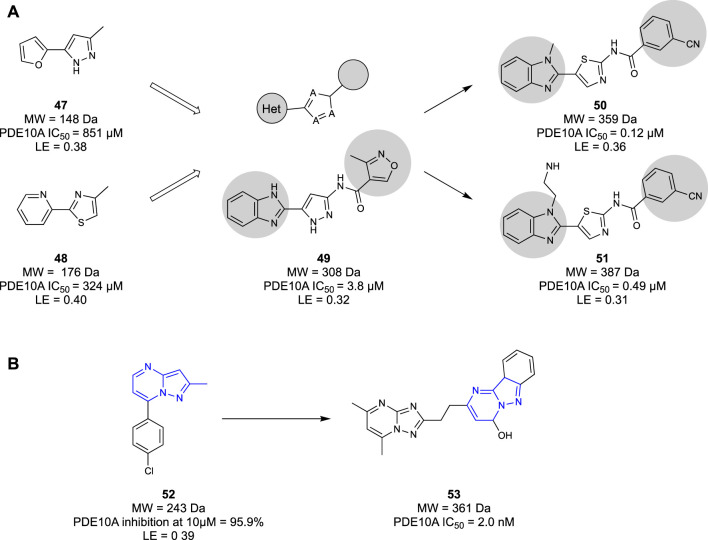
**(A)** Heterocyclic scaffold generated from fragment screen, with fragment hits **47** and **48** exemplifying the core structure. Compound **49** is the lead selected from the HTS candidates which was rapidly optimised into compounds **50** and **51**. **(B)** Fragment hit **52** and lead compound **53**.

AstraZeneca developed a PDE10A inhibitor borne of a dual fragment and conventional drug discovery campaign (Varnes et al., 2016). A HTS of the AstraZeneca corporate compound library against a PDE10A inhibition assay at 100 μM yielded 11,000 hits, at a high hit rate of 5%. Hits from this library that had structural similarities to known PDE inhibitors were discounted to ensure that any novel hits had a favourable selectivity profile for the PDE10A receptor. They identified 5,328 compounds as possible starting points for lead development. To sift through the hits identified, a low molecular weight fragment screen was initiated to narrow down the possible HTS leads. Some 3,000 fragments from the corporate compound library were screened, yielding 414 hits at a high hit rate of 14% at 100 μM. From this screen, an atypical group of fragment hits were identified, exemplified by **47** and **48**, which had different structural features from other hits. These unique fragment hits were then evaluated in an *in silico* docking study to indicate binding modalities unique to these structures. This *in silico* study highlighted a key scaffold, a five-membered ring core connected to a heterocycle and aromatic/hydrophobic group (Figure 9A). This key scaffold was believed to be essential to the novel binding modality. Compounds from the HTS campaign were then compared to this key scaffold in the hopes of maintaining the novel binding modalities discovered. This filtered the potential leads from 5,328 to just 14 compounds and was even further refined to only one after structures with unfavourable physicochemical characteristics and false positives were discarded. Having narrowed the HTS hits down to the lead compound **49**, the scaffold was rapidly optimised to compounds **50** and **51** (IC50 = 0.12 μΜ and IC50 = 0.49 μΜ, respectively). Ultimately, the fragment screen used by AstraZeneca acted as a filter in their drug discovery program term “fragment-assisted drug discovery” (FADD). Their FADD campaign identified lead compound **49**, which would have been overlooked if the initial screening had only been undertaken through HTS. Compounds **50** and **51** serve as a starting point for the next generation of PD10A antagonists from AstraZeneca.

Chino et al. (2018) utilised a fragment-based technique to generate a novel pyrimido[1,2-*b*]indazole chemotype of PDE10A inhibitors. Fragments from an undisclosed in-house fragment library were screened in an x-ray crystallography assay, identifying fragments that co-crystallised in the receptor. A pyrazolopyrimidine core **52** (Figure 9B) was selected as a starting point from the screen as it was identified as interacting with a key Gln726 residue within the binding pocket However, it was seen that the fragment did not interact with a crucial Tyr693 residue that dictated PDEA10A selectivity identified in previous studies (Chappie et al., 2009; Verhoest et al., 2009). To remedy this, the fragment was grown to increase its synthetic tractability to make screening heterocycles to interact with the Tyr693 residue easier. The co-crystal structure of **52** bound to the PDE10A receptor showed that the 4-chlorophenol moiety had no valuable interactions in the binding pocket. Thus, SAR studies investigating substitution at the 7-position showed that substitution with a hydroxyl group had the greatest increase in potency. The desired Tyr693 residue interaction was then created through substitution at the 5-position of the pyrazolopyrimidine with heterocycles and alkyl chains of various sizes. Compound **53** showed the desired interaction with Tyr693, as well as no inhibition of CYP enzymes, low PGP efflux (net efflux ratio = 1.8), and high metabolic stability (0 mL/min/kg). Therefore, compound **53** was identified as a lead compound for future work in a new series of PDE10A inhibitors from Astellas Pharma.

#### 2.3.2 Catechol O-methyltransferase

Catechol O-methyltransferase (COMT) catalyses the degradation of neurotransmitters such as dopamine and epinephrine, stopping their natural biological activity. In regions of the brain that have low concentrations of these neurotransmitters, COMT activation can prevent effective communication between cells. Therefore, in disease states characterised by low concentrations of neurotransmitters, such as dopamine and epinephrine, COMT inhibition shows promise as a therapeutic target to alleviate symptoms. The inhibition of COMT is theorised to abate some of the cognitive impairments and negative symptoms seen in schizophrenia patients (Apud and Weinberger, 2007; Akhtar et al., 2020).

**FIGURE 10:**
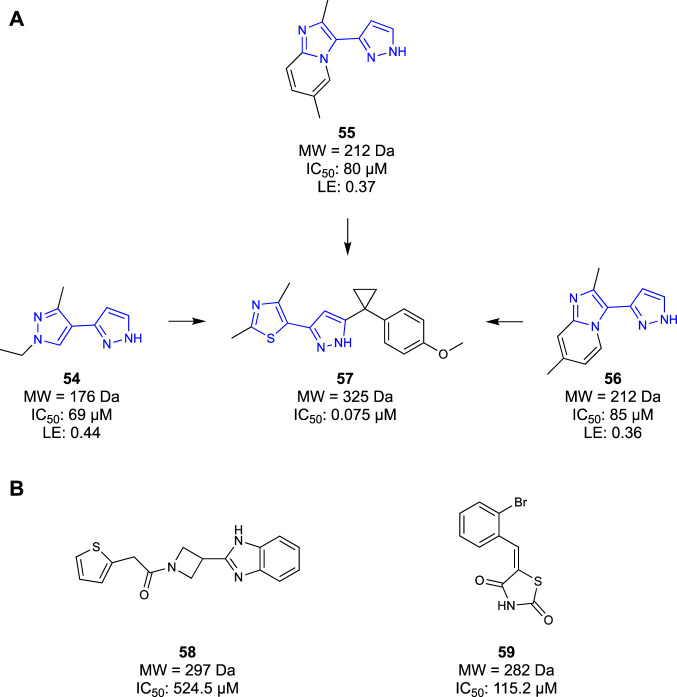
**(A)** COMT Fragments hits identified by Lerner et al. and the eventual lead compound synthesised. **(B)** KAT-II lead compounds identified by Jayawikrama et al.

COMT inhibitors have been successfully used in neurodegenerative disease as a therapeutic agent for treating Parkinson’s disease (Kiss and Soares-da-Silva, 2014). However, these COMT inhibitors were rapidly metabolised and peripherally restricted, serving to protect the liver, rather than permeating into the CNS. Similarly, previous generations of COMT inhibitors also suffered from poor pharmacokinetic properties, generally resultant of phenolic metabolism in the body (Tangphao et al., 1999; Wu et al., 2011). To distance themselves from these unfavourable molecules, Lerner et al. (2016) opted to take a fragment-based approach to identify a novel scaffold of COMT inhibitors. They set out to design competitive inhibitors of the co-factor S-adenosyl-l-methionine (SAM) binding pocket, thereby inhibiting COMT function. Initially, a fragment screen of 6000 Ro3 compliant molecules from an undisclosed library were screened in an SPR assay, yielding 600 hits. The identified fragment hits were then confirmed in a ten-point SPR concentration-response measurement, identifying 200 confirmed binders. The confirmed fragment binders were then reconfirmed again in a 1D ^1^H-NMR study before being characterised in a 2D ^1^H/^15^N HSQC study to validate the binding interactions. The IC_50_ of all 600 hits was then determined in an enzymatic fluorescence-based assay. The results of all four assays were combined and considered, yielding four compounds that performed well in all assays. Of these four hits, compounds **54**–**56** were selected as lead compounds with a high LE (Figure 10A) and moderate potencies with IC_50_ ranging from 69 μM to 85 μM. It was noted that one of the four fragments was similar to known COMT inhibitors, so a co-crystallisation study was undertaken to confirm that the identified fragments bound in the SAM pocket. Furthermore, the co-crystallisation study provided detailed binding interactions that would inform the SAR, aiding in the optimization process. Optimisation efforts produced compound **57** with a potent COMT inhibitor (IC_50_ = 0.075 μM) Ro3-compliant lead molecule for future work. Ultimately, Lerner et al. (2016) developed a novel chemotype and subsequent lead molecules to target the SAM pocket of the COMT enzyme. Their fragment-based approach ensured that they could tailor their molecules to increase affinity for the SAM pocket whilst avoiding the metabolism that had plagued previous generations of COMT inhibitors.

#### 2.3.3 Kynurenine aminotransferase

Kynurenine aminotransferase-II (KAT-II) is an enzyme involved in the metabolism of tryptophan. KAT-II converts kynurenine into kynurenic acid (KYNA), the latter being both an antagonist of N-methyl-d-aspartic acid (NMDA) and acetylcholine receptors (Plitman et al., 2017). The antagonism of both receptors, a result of KYNA, is linked to the cognitive impairments seen in schizophrenia (Koshy Cherian et al., 2014). Previous generations of KAT-II antagonists have demonstrated reduction in KYNA levels, whilst increasing levels of neurotransmitter signalling exhibited the potential to ameliorate cognitive impairments seen in schizophrenia (Pellicciari et al., 2006; Wu et al., 2014; Nematollahi et al., 2016).

Jayawickrama et al. (2018) utilised a fragment-based approach to identify a new class of KAT-II inhibitors and gain a new understanding of KAT-II inhibition. Initially, 1,000 compounds were screened from a structurally diverse in-house library, with an average molecular weight of 223 Da. Fragments were evaluated in an SPR assay, yielding 41 promising compounds that would undergo a concentration–response SPR to eliminate false positives. Only 18 compounds remained after the assay, all having confirmed affinity for the KAT-II binding site. The 18 compounds were screened in an HPLC inhibition assay wherein only three compounds had inhibitory effects. One of the three compounds was discarded due to similarities to another fragment, ultimately yielding two fragments as a base for further optimisation—**58** and **59** (Figure 10B). These two fragments also served as benchmarks for a computational study, evaluating the key binding interactions needed for potent KAT-II receptors. Jayawickrama et al. (2018) successfully discovered two new chemotypes of KAT-II inhibitors, serving as leads for the development of novel therapeutics.

### 2.4 Huntington’s disease

Huntington’s disease (Huntington’s) is a neurodegenerative disease that causes the progressive decline of both cognitive and motor function, amongst other psychiatric and behavioural issues (Bates et al., 2015). Huntington’s is characterised by the expansion of a cytosine–adenine–guanosine repeat on the huntingtin gene (HTT). Huntington’s is pathologically defined by this mutant huntingtin (mHTT) gene (Bates et al., 2015). Following the onset of symptoms, the progression of the disease is ultimately fatal, with a median survival time of 15–18 years (Caron et al., 1993). Currently, there are no therapies available to alleviate the symptoms or slow the progression of Huntington’s. Ko et al. (2001) identified that antibody MW1 effectively binds to both HTT and mHTT. They suggested that MW1 binding to mHTT may prevent protein aggregation and serve as a therapeutic target for Huntington’s (Ko et al., 2001). In 2007, a crystal structure of MW1 bound to mHTT was isolated by Li (2007) that would serve as the basis of future FBDD campaigns.

**FIGURE 11:**
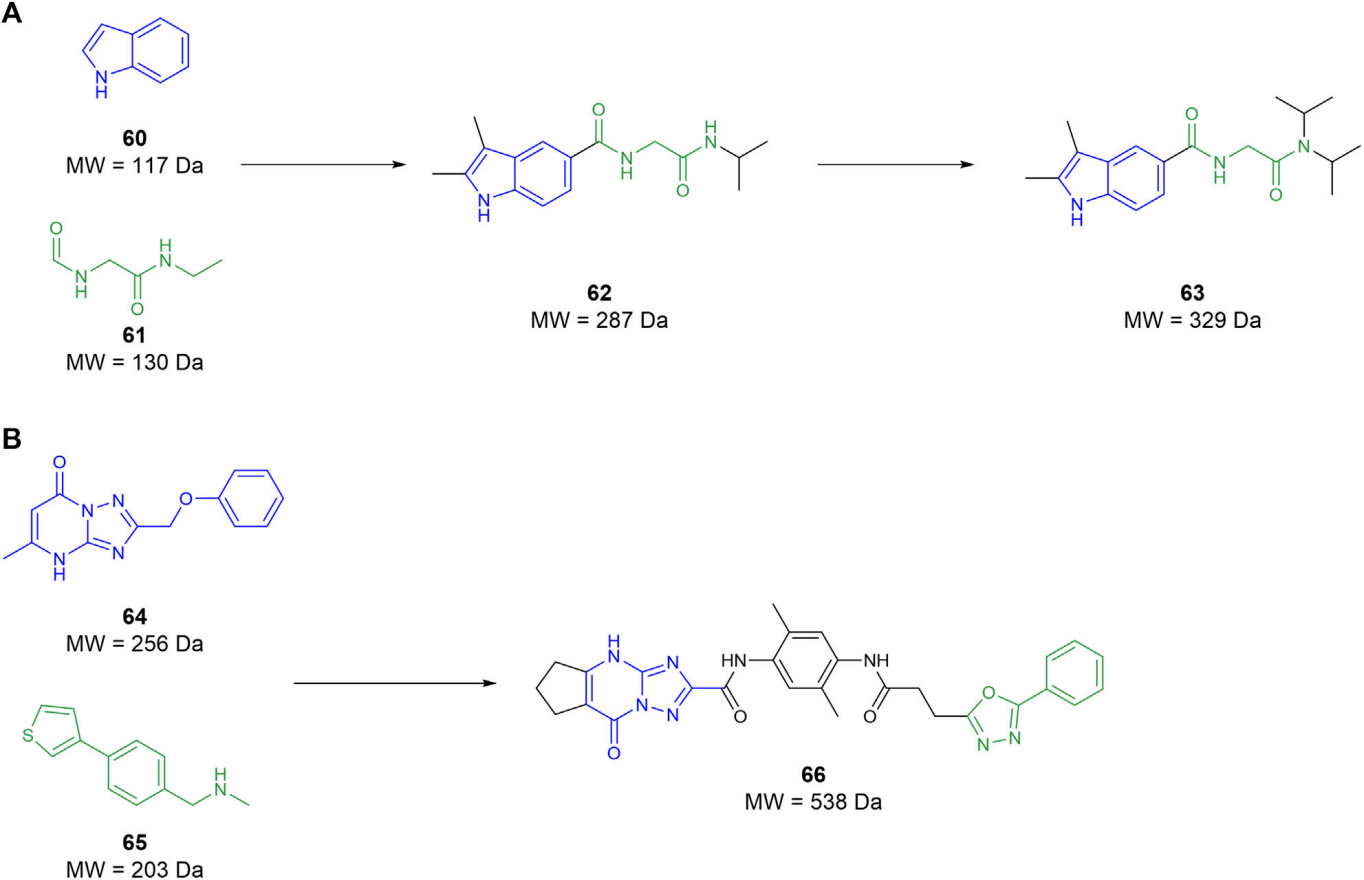
**(A)** Workflow from Galyan et al. to develop a preclinical candidate GLYN122 (**63**). **(B)** Lead molecule for glioblastoma from Kegalman et al.

In 2022, Galyan et al. (2022) conducted an FBDD campaign informed by the crystal structure previously identified Li (2007). Initially, the known mHTT crystal structure was simplified down to a collection of surface feature points. This simplified structure was then compared to the protein-database bind (PDBbind) containing the crystal structures of 240,013 interactions between chemical fragments and protein environments. When a surface from the PDBbind database matched the surface feature points from the mHTT structure, the fragment from the former was mapped onto the latter. This yielded 82 fragments that covered a reasonable portion of the protein–protein interaction between the MW1 antibody and the mHTT protein. These fragments were then used to screen 12 million compounds *in silico* from catalogues of unnamed commercial vendors. Compounds identified from the chemical vendors were considered compatible if they contained a substructure with atoms matching at least two-thirds of the atoms of the 82 selected fragments. Compounds that contained the same fragment substructure were selected based on size, with the larger molecules being discarded. This filter identified 2,937 suitable compounds to be examined, of which fragments **60** and **61** were notable upon re-examination as they comprise compound **62** (Figure 11A). The compounds that passed were then docked against the mHTT structure and ranked based on predicted binding energy. Compounds that shared a surface overlap with another fragment of over 66% were filtered out, keeping only the fragment with the best predicted binding ability. This reduced the suitable compounds from 2,937 to 67, wherein 40 compounds were purchased from vendors to begin testing. The purchased compounds were screened using SPR, identifying four compound hits that showed a concentration-response of binding, among which compound **62** was identified. Commercially available derivatives of the four initial hits that comply with Lipinski’s Ro5 and a Tanimoto similarity score of 80% were purchased and screened using SPR. The SPR screen showed that two derivatives had improved potency relative to the initial four hits identified. The two second-round hits were then used to identify similar analogues using the same similarity metrics defined above. However, this time, the filter included a polarity metric (TPSA < 75 Å) to improve BBB permeability, identifying 56 more candidates. The third-round SPR screen identified 20 compounds with improved affinity relative to the initial first-round hits. These compounds were then screened in a PAMPA BBB permeability assay in which all compounds were seen to be BBB-permeable. The top eight compounds from the PAMPA assay were then tested in an mHTT clearance assay, selecting compound **63** as a hit for further *in vivo* testing. This showed that **63** attenuated motor deficits and reduced mHTT expression in transgenic mice. However, the doses required to do so were too high for translation into human trials. These studies suggest that **63** is a good preclinical candidate for the treatment of Huntington’s, but its potency must be improved to reduce the dose needed for human administration. Galyan et al. (2022) effectively demonstrate that fragment-assisted drug discovery and SAR by catalogue can rapidly improve the potency of drug candidates without the need for synthetic medicinal chemists.

### 2.5 Glioblastoma

Glioblastoma multiforme (GBM) is currently an incurable disease with an average survival time of 15 months and only 5.5% of patients surviving 5 years post-diagnosis. GBM is a fast-growing, aggressive brain tumour that affects the brain and spine and generally develops from glial cells. Melanoma differentiation-associated gene 9 (*mda*-9) is implicated in the invasion and metastatic signalling of GBM, amongst other cancers (Das et al., 2019). MDA-9 stimulates the invasion, angiogenesis, and tumour progression of GBM. Consequently, the inhibition of MDA-9 expression is theorised to decrease GBM invasion and improve patient outcomes (Das et al., 2019). A key characteristic of MDA-9 is the presence of two tandem PDZ domains (PDZ1 and PDZ2). These PDZ domains enable protein–protein interactions that stimulate pro-invasive signalling in GBM (Kegelman et al., 2017).

Kegelman et al. (2017) aimed to develop an MDA-9 inhibitor to prevent invasion by and metastasis of GBM as a supplement to radiotherapy. Utilising a ^15^N NMR-based screening, 5,000 fragments were tested with a ^15^N-labelled PDZ1/2 tandem domain from MDA-9. The screening yielded two fragment hits, **64** and **65**, which both interacted at the PDZ1 domain, while no suitable fragments interacted at the PDZ2 domain. A subsequent docking campaign suggested that both fragments needed substantial elaboration to effectively fill the binding pocket. The two fragments were linked, and the resulting compound was optimised through subsequent SAR studies yielding compound **66** (Figure 11B). This compound possesses micromolar affinity for PDZ1, with very slow clearance (T_1/2_ ≥ 9 h) and, surprisingly, the ability to cross the BBB. Patients suffering from GBM have a defective blood–brain barrier, meaning that antigens that do not typically permeate the BBB can still pass through. As compound **66** is not traditionally CNS permeable, (MW > 450), it can exploit the imperfect BBB to exert its therapeutic effect. Subsequent *in vivo* assays suggest that **66** is a radiosensitizer and is useful as an adjunct to radiotherapy. Ultimately, Kegelman et al. (2017) utilised FBDD to create a new class of small molecule drugs to target GBM and other advanced, targeted brain cancers.

### 2.6 Neuroinflammation

Neuroinflammation is an incredibly complex phenomenon that can be attributed to a variety of stimuli. As neuroinflammation can be due to so many causes, the context of the inflammation must be considered. Acute neuroinflammation can be neuroprotective, repairing or rebuilding the brain post injury (DiSabato et al., 2016). However, chronic neuroinflammation is almost always detrimental to the brain (DiSabato et al., 2016). Given the delicate environment of the CNS, developing effective therapeutics for the treatment of neuroinflammation is extremely challenging.

**FIGURE 12:**
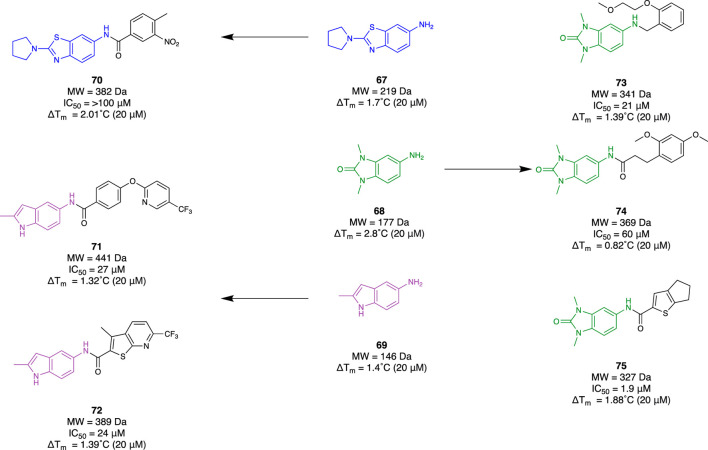
Fragment hit identification to lead molecules for BRD4 from Borysko et al.

Bromodomain and extra-terminal domain (BET) protein has been implicated as playing a major role in the transcriptional regulation of the inflammatory response. There are four mammalian members (BRD2, BRD3, BRD4, and BRDT). Understanding the mechanistic behaviour of BET proteins has been of great importance in revealing potential novel therapeutics for neuroinflammation. Borysko et al. (2018) demonstrated a fragment-based approach by using “SAR by catalogue” for BRD4. They employed an Enamine Ro3-compliant fragment collection for 7450 compounds. These underwent solubility confirmation with DMSO at 200 mM, a 2D fingerprint-based diversity filtering followed by visual inspection, resulting in a group of 3,695 fragments. Subsequent TSA was conducted to determine their mean thermal shifting value (ΔT_m_) using a recombinant, truncated His-tagged bromodomain 1 of BRD4. Observing fragments exhibiting positive thermal shifting and negative thermal shifting, 79 primary hits were selected (48 positive and 31 negative) which included fragments **67–69** (Figure 12). Utilising this set of fragments, a substructure search was completed within the Enamine *REAL* database to identify hit molecules that could be synthetically accessed within 3–4 weeks to improve time efficiency for their SAR study. Through physicochemical evaluation, fingerprint-based diversity selection, and visual inspection, 3,200 compounds derived from the group of hit fragments were selected for screening and classed as “active” compounds. The same process was repeated with the class of “non-hit” fragments to also obtain a collection of 3,200 compounds that were grouped as “non-active” compounds. Following this, a random selection of 3,200 compounds was made within Enamine’s stock screening collection with MW and cLog*P* comparable to the “active” compounds. TSA was conducted with these three groups to obtain 61 hits (39 from the “active” group, 10 from the “non-active” group, and 12 from the “random” group). Subsequently, a fluorescence resonance energy transfer assay against BRD4 found 18 compounds that exhibited at least 50% inhibitory activity. Six molecules, **70–75**, derived from the group of “active” fragments saw greater than 60% inhibitory activity whilst also having a positive mean thermal shift value (Figure 12). The workflow of this study demonstrated how a fragment-based campaign with SAR by catalogue can be an efficient pipeline for early drug discovery. Not only was a group of active molecules for BRD4 identified, but it demonstrated the enhanced efficiency of utilising FBDD to first identify suitable scaffolds.

## 3 Perspective

FBDD has been effectively utilised to develop hit and lead molecules, both independently and as an adjunct to traditional drug design methods. Each of the examples discussed follows the workflow of identifying a hit/lead molecule and subsequent optimisation using knowledge gained throughout the FBDD process. This results in a more streamlined process of fragment elaboration and, therefore, a quicker turnaround time in drug development.

However, like all methods, FBDD is still bound by limitations that prevent it from being the gold standard of the drug discovery process. As demonstrated through these studies, FBDD relies heavily on x-ray crystallography to provide data on the binding modes of the fragment to subsequently direct synthetic elaboration towards a lead molecule. Understanding where each fragment binds and what type of interaction is responsible for the binding event is crucial to constructing a molecule that complements the domain of the binding pocket. Unfortunately, this poses a disadvantage to certain targets that are “un-crystallisable” or difficult to crystallise, such as membrane-bound receptors (Carpenter et al., 2008). The flexibility and instability of these proteins lead to issues with expression, solubility, purification, and subsequent crystallisation. As a result, membrane-bound targets that cannot be crystallized must use predicted structures rather than the actual target, effectively eliminating the advantages of FBDD.

It is also challenging to employ FBDD to target protein misfolding-derived neurodegenerative diseases. As FBDD relies on a target with a known morphology, processes that have an unknown or ever-changing morphology are not suitable in fragment-based campaigns (Sweeney et al., 2017). For these cases, a traditional target-specific screen may be a more appropriate approach for developing therapeutics. This is highlighted in Aβ, where the pathogenic species initially proposed in AD was fibrils and plaques but now is believed to be aggregation intermediates such as oligomers and soluble protofibrils (Hampel et al., 2021). Chaperones, such as heat shock proteins (Hsp), and particularly Hsp70, are a popular alternative target class for therapeutic intervention in protein misfolding diseases. Although Hsps are considered “druggable” targets, their binding pockets are generally located at a protein–protein interface (PPI), which is extremely challenging to drug. PPIs comprise many small interactions called “hot spots”, wherein a small molecule can bind. Fortunately, FBDD is inherently good at identifying and mapping these hot spots. For this reason, fragment-based approaches have achieved success in regulating PPIs for human disease therapy, with examples such as *venetoclax* and *sotorasib* as FDA-approved PPI modulators.

Furthermore, fragment-based studies have consistently been limited to target-based studies. Unfortunately, the last 150 years has demonstrated that only 9.4% of small-molecule drugs have been identified through a target-based approach (Sadri, 2023). When exploring the list of FDA-approved drugs, many exhibit activity at more than one target, suggesting that the therapeutic effect may be the result of more than just the modulation of one key target. For example, physical changes to the brain which lead to AD include amyloid plaques, neurofibrillary tangles, and inflammation (Selkoe, 1991). Targeting the genesis of these physical changes by inhibiting Aβ generation, promoting Aβ clearance from the brain, or inhibiting the formation of tau tangles all aim to slow the inflammatory response (Yoon and AhnJo, 2012). However, we now know that these are all examples of treating a manifestation of the disease, rather than the underlying cause. This is not to say that target-based approaches cannot yield a drug. Such approaches are hypothesis-driven; however, phenotypic studies rely on measures of responses (Swinney, 2013). Nevertheless, the ambiguity of where to target and the nature of the target creates a challenge during hit identification and optimisation when utilising FBDD. Although conventional studies such as HTS suffer from similar limitations, they excel in this area, such as the HTS library of drug-like molecules which can produce a tangible response in a phenotypic screening which, unfortunately, is impossible for fragments.

Most FBDD campaigns utilise a combination of various biophysical screenings to determine and validate fragment hits. These methods generally provide a binding affinity value which is improved as the hits are elaborated. However, binding affinity and binding mode are properties that do not always correlate linearly with a target response. Unfortunately, due to the small size of the fragment, biochemical data may be difficult to obtain. To identify any correlation between binding affinity value and activity, some studies, such as the examples discussed earlier, utilise a biochemical assay as soon as possible to obtain values in the milli- to micromolar range, or to identify maximum inhibition at a particular concentration. This will not only probe the target more efficiently but could potentially improve the physicochemical properties of the molecule by omitting unnecessary customisation.

The well-considered curation of fragment libraries is advantageous for efficiently sampling chemical space. Many libraries, especially those highlighted earlier, demonstrate a lack of sp^3^-rich compounds. Drug discovery has long had issues with tackling the spatial arrangement of a pharmacophore group to enhance the properties of the molecule (Meutermans et al., 2006). Sp^3^ fragments are frequently omitted from libraries to prevent synthetic inaccessibility from being a barrier to efficient lead compound development (Caplin and Foley, 2021). Therefore, the utilisation of sp^3^ fragments can build greater diversity into libraries whilst also exploring new and potentially chiral chemical space. Consequently, sp^3^-rich molecules are generally considered less rigid, being able to cover a broader chemical space than their typically planar aromatic counterparts. This lack of rigidity and planarity can be advantageous in drug design, allowing a molecule to more broadly fill out a binding pocket, exemplified by carbohydrates and spirocycles (Meutermans et al., 2006; Sveiczer et al., 2019). Limiting the scope of potential fragments can hinder the development of superior lead molecules.

FBDD represents growth towards a new age of drug discovery. More than a complementary approach, it has emerged as a pivotal tool to overcome limitations that have proven difficult with more conventional methods. By identifying optimal scaffolds followed by a promising lead with minimal elaboration, FBDD is capable of fulfilling the stringent criteria for developing CNS-tailored small molecules. It reveals optimal interactions to offer crucial insights for designing superior molecules. FBDD has the potential to reshape the drug discovery landscape, setting new standards for pharmaceutical development.
